# Ecologies, synergies, and biological systems shaping human milk composition—a report from “Breastmilk Ecology: Genesis of Infant Nutrition (BEGIN)” Working Group 2

**DOI:** 10.1016/j.ajcnut.2022.11.027

**Published:** 2023-05-10

**Authors:** Jennifer T. Smilowitz, Lindsay H. Allen, David C. Dallas, James McManaman, Daniel J. Raiten, Mary Rozga, David A. Sela, Antti Seppo, Janet E. Williams, Bridget E. Young, Michelle K. McGuire

**Affiliations:** 1 Department of Food Science and Technology, University of California Davis, Davis, CA, USA; 2 Foods for Health Institute, University of California Davis, Davis, CA, USA; 3 United States Department of Agriculture, Agricultural Research Service, Western Human Nutrition Research Center, University of California Davis, Davis, CA, USA; 4 Nutrition Program, College of Public Health and Human Sciences, Oregon State University, Corvallis, OR, USA; 5 Division of Reproductive Sciences, University of Colorado, Aurora, CO, USA; 6 Pediatric Growth and Nutrition Branch, *Eunice Kennedy Shriver* National Institute of Child Health and Human Development, National Institutes of Health, Bethesda, MD, USA; 7 Evidence Analysis Center, Academy of Nutrition and Dietetics, Chicago, IL, USA; 8 Department of Food Science, University of Massachusetts, Amherst, MA, USA; 9 Department of Microbiology and Physiological Systems, University of Massachusetts Medical School, Worcester, MA, USA; 10 Department of Pediatrics, Division of Allergy and Immunology, University of Rochester School of Medicine and Dentistry, Rochester, NY, USA; 11 Department of Animal, Veterinary and Food Sciences, University of Idaho, Moscow, ID, USA; 12 Margaret Ritchie School of Family and Consumer Sciences, University of Idaho, Moscow, ID, USA

**Keywords:** human milk, breast milk, composition, ecology, systems biology, nutrition

## Abstract

Human milk is universally recognized as the preferred food for infants during the first 6 mo of life because it provides not only essential and conditionally essential nutrients in necessary amounts but also other biologically active components that are instrumental in protecting, communicating important information to support, and promoting optimal development and growth in infants. Despite decades of research, however, the multifaceted impacts of human milk consumption on infant health are far from understood on a biological or physiological basis. Reasons for this lack of comprehensive knowledge of human milk functions are numerous, including the fact that milk components tend to be studied in isolation, although there is reason to believe that they interact. In addition, milk composition can vary greatly within an individual as well as within and among populations. The objective of this working group within the Breastmilk Ecology: Genesis of Infant Nutrition (BEGIN) Project was to provide an overview of human milk composition, factors impacting its variation, and how its components may function to coordinately nourish, protect, and communicate complex information to the recipient infant. Moreover, we discuss the ways whereby milk components might interact such that the benefits of an intact milk matrix are greater than the sum of its parts. We then apply several examples to illustrate how milk is better thought of as a biological system rather than a more simplistic “mixture” of independent components to synergistically support optimal infant health.

## Introduction

The “Breastmilk Ecology: Genesis of Infant Nutrition (BEGIN)” Project was designed to *1*) examine the ecology of human milk, based on the supposition that human milk represents a complex biological system that interacts with both the internal (biology and health of the lactating person, human milk matrix, and impact of and on the breastfed infant) and external (social, behavioral, cultural, and physical) environments (see Text Box 1 for core concepts and terms); *2*) explore the functional implications of this ecology for both biological parent and their infant; and *3*) explore the ways in which this emerging knowledge can be studied and expanded via a targeted research agenda and translated to support the community’s efforts to ensure safe, efficacious, equitable, and context-specific infant feeding practices in the United States and globally.

Text Box 1 **Core concepts and terms.**

• In the context of this paper, “ecology” is defined as a complex biological system and its interactions with its environment. In this case, the complex system is human milk composition and its inherent biology, and the environment consists of parental and infant inputs and the influence of their respective internal and external environments.
• With due recognition of the need to be observant of issues of gender identity/neutrality and to improve precision, to the extent possible, for the purposes of the papers described herein, we will use gender-neutral terminology where appropriate (e.g., lactating parent or person, etc.) to reflect the reality that not all who lactate identify as female. The term “lactating parent” respects and recognizes those who may have been born as female but do not identify as such and other gender-relevant contingencies. In situations where primary data are reported (studies or analyses), we will refer to the population as specified (e.g., “the study evaluated 250 lactating mothers”). Moreover, rather than using terms such as “maternal” or “maternal milk,” we will use the terms such as “birthing parent” throughout the report as appropriate as they accurately reflect the biological nature of the birthing parent-infant dyad.
• “Human milk” refers to milk produced by lactating parents and includes both *1*) breastmilk produced by a parent for their infant and fed directly to infants via the breast or expressed by the lactating parent and then fed to the infant and *2*) donor or banked human milk produced by lactating persons that is either donated to human milk banks or fed to infants other than their own child.

Alt-text: Text Box 1

The overarching conceptual framework and description of the Project are presented in the BEGIN executive summary, the first of 6 manuscripts of this supplement [1]. We hope that the subsequent manuscripts in this supplement presenting the findings of all of the individual thematic BEGIN Working Groups (WGs) are read as a series that represents a continuum of thought reflecting a larger conceptual view of how we can move this important research and public health agenda forward.

Specifically, the BEGIN Project was accomplished via the constitution of 5 thematic WGs charged with addressing the following themes: *1*) parental inputs to human milk production and composition; *2*) the components of human milk and the interactions of those components within this complex biological system; *3*) infant inputs to the matrix, emphasizing the bidirectional relationships associated with the breastfeeding dyad; *4*) the application of existing and new technologies and methodologies to study human milk as a complex biological system; and *5*) approaches to translation and implementation of new knowledge to support safe and efficacious infant feeding practices. This paper represents the results of the deliberations of WG 2.

### A Conceptual Framework: “Nourish, Protect, Communicate”

**FIGURE 1:**
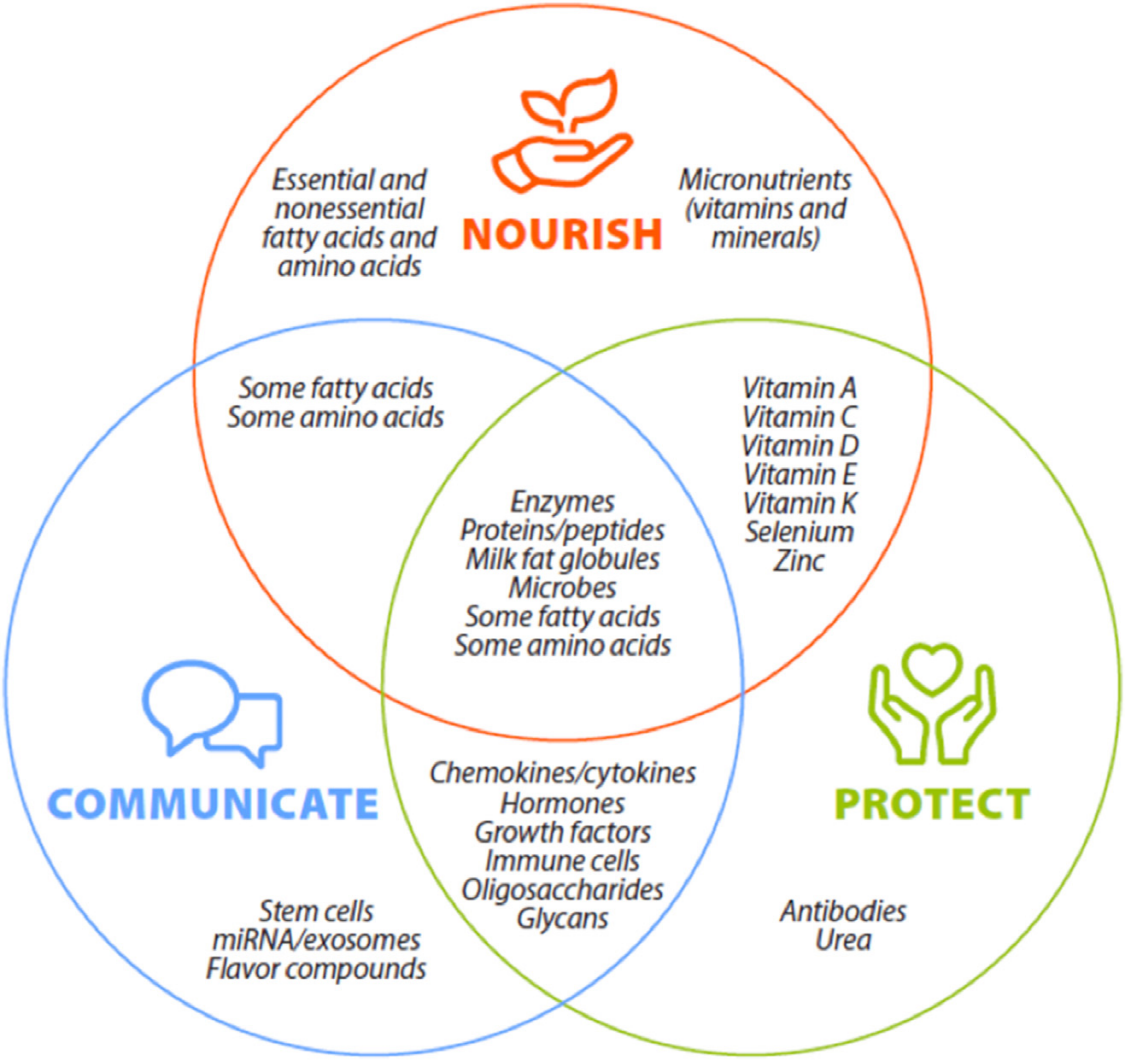
Human milk constituents provide myriad benefits to infants, including nourishment (supporting physical growth, development, and metabolism), communication (relaying complex information as to how to best thrive in a particular environment and culture and with a specific genetic background), and protection (shielding the infant from infection and supporting the infant when the infant’s immune system is naive). Whereas some components have a single function, most play multiple roles.

Human milk is a remarkable consequence of selective evolutionary pressures combined with short- and long-term exposures that collectively provide a context-specific, complete, and complex diet during early life. Although human milk certainly delivers highly bioavailable macronutrients and micronutrients, it provides much more than nourishment. Indeed, milk also delivers other biologically active components such as immunoglobulins, peptides, hormones, leukocytes, microbes, complex carbohydrates, milk fat globule membranes (MFGMs), and intracellular vesicles that individually and coordinately support, protect, and guide infant growth and development. Moreover, human milk delivers many of these biomolecules in the form of complex, supramolecular structures that synergistically nourish, protect, and communicate information to the infant. These overarching and overlapping functions (i.e., nourish, protect, and communicate) of human milk components are illustrated simplistically in Figure 1.

Perhaps the best understood function of human milk is delivering nutrients that provide energy and are basic building blocks needed for growth and development—in other words, human milk nourishes the infant. These nutrients are generally categorized as essential, conditionally essential, or nonessential based on whether they must be consumed from the diet. In addition to the classic nutrients and nutrient-like components, however, milk contains many immunologically active elements (including host cells) that directly and indirectly protect the infant during critical windows of immune system development. Milk also contains a host of potentially important proteins, peptides, complex lipid and glycan structures, and microbes that may contribute to infant health via colonizing the gastrointestinal (GI) tract, producing useful metabolites, and communicating important information to the infant about endemic microbial conditions.

Historically, individual researchers have tended to study 1 or 2 minerals, a set of related vitamins, or a particular type of macronutrient or macromolecule. Although this reductionist approach helped classify nutrients into elementary functional categories in the 19th and 20th centuries, facilitate the prevention and cure of nutrient deficiency diseases in the mid-20th century, and provide necessary fundamental knowledge related to human milk composition and its impact on infant health, this approach does not adequately address the complexity of human milk biomolecules that synergistically target multiple complex systems in the infant. A systems biology approach using the lens of ecology, combined with the diverse toolsets and multidisciplinary methods, offers a more fruitful opportunity to identify mechanisms driving variation in milk components and their effect on infant health.

In this report, we first briefly review human milk composition and its importance in nourishing, protecting, and communicating complex information to the infant. In this section, we also highlight some of the many research questions that need to be addressed to understand the impacts of milk components (both independently and synergistically) on infant health and wellbeing. We then provide a framework by which human milk components can be thought of as interacting within both the milk matrix and broader ecosystem such that “the whole is greater than the sum of its parts.” The reader is directed to other WG reports in this series for more detailed information on factors impacting variation in milk composition [2, 3], optimal study designs for exploring milk as a biological system [4], and a translational framework tailored to human lactation and infant feeding [5].

### Overview of Milk Components, Variation, and Importance to Infant Health

**FIGURE 2:**
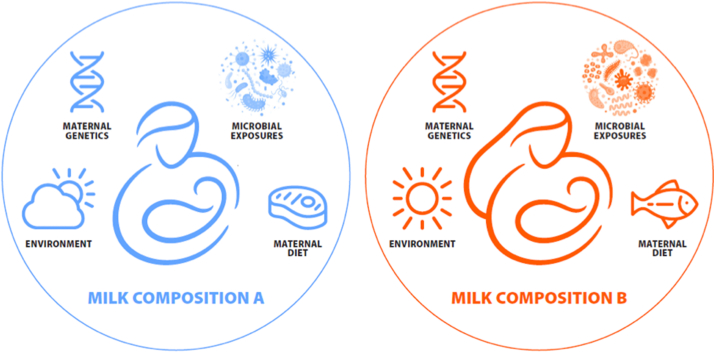
Milk composition varies greatly among individuals and populations, and it is possible (albeit understudied) that this variation might differentially support optimal health and survival of infants living in varying ecological contexts, including those related to lactating parent/infant genetics, food/nutrient availability, and environmental exposures (e.g., pathogens). This concept of a shift in “normal” to support health in a particular ecosystem is referred to as ecohomeorhesis (9).

Many human milk components are highly variable and dynamic, and this fact can confound attempts to describe their functions scientifically. Moreover, there is growing evidence that what is typical in terms of milk composition varies across regions and cultures [6, 7] and that these differences (some of which likely resulted from selective pressures [8]) might be important for infant survival and wellbeing. This concept of a variable “normal” milk composition to support health in a particular ecology has been referred to as eco-homeorhesis [9], which we use throughout this manuscript. The term eco-homeorhesis describes customized adjustments of homeostasis in a dynamic system to maximize health and/or fitness within a particular ecosystem. Eco-homeorhesis differs from homeorhesis, which describes adjustments of homeostasis to support a particular physiologic state such as pregnancy or lactation [10]. When studying milk as a biological system, it is therefore critical to consider shifts in normal milk composition that may occur in response to the ecosystem (eco-homeorhesis) and also in response to the process of lactation (homeorhesis). We posit that the concept of eco-homeorhesis is particularly pertinent to understanding variation in human milk composition, especially across geographic locations and cultures. Indeed, there may not be a one-size-fits-all construct for human milk composition, with eco-homeorhetic differences contributing to context-specific fitness (Figure 2). Aside from the documented instances of nutrient deficiencies, however, little is known about if and how milk component variation is related to infant health.

### Micronutrients

At least when produced by well-nourished individuals, human milk contains all the essential micronutrients (vitamins and minerals) needed by infants. Micronutrients support infant health similarly to how they support adult health. Additionally, variation in the concentrations of some micronutrients in human milk may communicate information (e.g., chronic nutrient availability) on the environment to the infant. Myriad factors are known to impact the variation in the concentrations of micronutrients, with maternal diet and nutritional status being particularly important. Note that, for the purposes of this paper, we use the term “maternal” where appropriate to modify factors (e.g., diet, genetics) associated with the lactating/birthing parent so as to clearly distinguish the birthing parent from the other parent.

A useful categorization of milk micronutrients is into one of the 2 following groups: those for which concentrations in milk are affected by the maternal nutrient status and diet or supplementation (e.g., thiamin, riboflavin, niacin, biotin, pantothenic acid, vitamin B6, vitamin B12, vitamin C, vitamin A, vitamin D, vitamin E, vitamin K, choline, iodine, and selenium) and those for which the milk concentrations are independent of the maternal nutrient intake and/or status (e.g., folate, calcium, iron, copper, zinc, sodium, chloride, potassium, phosphorus, magnesium, manganese, fluoride, chromium, and molybdenum), the latter leading to the depletion of maternal body stores when dietary intake is inadequate and/or inadequate concentrations in milk regardless of maternal intake [11, 12]. In addition, heat treatment (e.g., pasteurization) commonly used in donor milk banks can reduce concentrations of some micronutrients (e.g., calcium, phosphorus, folate, and vitamin C [13, 14]); whether this impacts infant health has yet to be elucidated. There is also circadian variation in the concentrations of milk micronutrients, being related to dietary consumption over the day and also with milk lipid content [15]. Very little is known about whether variation, regardless of its driver, in concentrations of micronutrients in milk produced by well-nourished individuals within a given geographic or cultural context is associated with variable health outcomes in infants. Similarly, whether differences in milk nutrient concentrations among populations support customized, optimal early-life fitness for a particular context is not known. As with all milk components, there remain numerous fundamental and applied research gaps related to micronutrients. Some of these research gaps are listed in text boxes throughout this manuscript. The potential for functional interactions among micronutrients and microbes found in human milk is highlighted in a later section of this report (Text Box 2).

Text Box 2 **Micronutrients in human milk: selected research gaps.**

• What are the cellular mechanisms whereby micronutrients are incorporated into milk, and what genetic, physiologic, and environmental factors are related to their concentrations in milk?
• Is variation in the concentrations of micronutrients and macronutrients in human milk produced by well-nourished individuals within a given geographic or cultural context associated with health outcomes in infants? What about variation among individuals across different geographic or cultural contexts?
• What are the overarching implications of food fortification and supplementation on human milk micronutrient content and infant health, and what are the consequences for developing dietary recommendations for infants?
• Are differences in human milk micronutrient concentrations among populations examples of ecohomeorhetic shifts to support optimal early-life fitness for a particular context?
• Do changes in micronutrient concentration due to pasteurization and cold storage influence infant health and wellbeing?
• Is the chronobiology of the micronutrients that vary over a 24-h period important for infant health and wellbeing?

Alt-text: Text Box 2

### Macronutrients

More than 250 million y of evolutionary pressure on lactation have resulted in the production of human milk containing an array of macronutrients that nourish, protect, and play a role in communication between the lactating parent and infant [16]. The most abundant macronutrient in human milk is water, which comprises ∼87%–91% of milk’s weight (dependent on the lactation stage) and is critical not only as a solvent for milk’s other constituents but also for basic hydration for the infant. The volume of water secreted in milk is primarily driven by lactose, which acts in coordination with infant demand as a major driver of milk volume [3]. Details regarding the energy-yielding macronutrients, their variability, and their importance to infant health are described briefly here.

#### Lactose

The disaccharide lactose is the second most abundant nutrient in human milk (∼70 g/L), and is instrumental in nourishing infants by providing a portion of their energy needs and by regulating the water content of milk. Lactose might also protect the infant from enteric pathogens by selectively promoting the growth of lactose-utilizing bacteria in the infant’s GI tract. Milk’s provision of lactose is accompanied by the production of lactase in the infant’s intestinal brush border membrane that digests this carbohydrate during infancy and childhood. After secretory activation, lactose concentration is relatively stable in human milk, not seemingly influenced by factors such as the lactation stage [17], time of day, left or right breast [17], fore vs. hind milk [18], and maternal diet [19]. Nonetheless, lactose concentrations have been shown to vary by prematurity [20] and global context [21], and the source of this variation is not understood. Lactose is relatively stable to freezing but is slightly reduced by pasteurization [22], [23].

#### Lipids and milk fat globules

Collectively, lipids are the next most abundant component in human milk (30–50 g/L), delivering nourishment, protection, and communication to the infant. Lipids in human milk are delivered as milk fat globules (MFGs) that range in 3 orders of magnitude in size from less than 200 nm to over 15 μm [24]. MFGs contain a nonpolar lipid core (e.g., triglycerides) surrounded by a complex trilamellar membrane composed of polar lipids (e.g., phospholipids, sphingomyelin, cholesteryl esters, and retinol esters) and membrane-bound proteins derived from the mammary epithelial cell’s own MFGM. This distinct structure uniquely specializes MFGs to be transported in the aqueous environments of both milk and the GI tract of the breastfed infant. Fatty acids found in the lipid core provide most of the energy needed for exclusively breastfed infants as well as the essential and conditionally essential fatty acids required for infant growth and development. The MFGM also delivers lipid-soluble signaling molecules such as prostaglandins, which may have roles in supporting the GI tract of the newborn (e.g., motility and mucin secretion). Other nonessential lipids found in the MFGM (e.g., free cholesterol and sphingolipids) are critical for membrane integrity and in scaffolding for cell–cell recognition and signaling. Additionally, glycolipids, found nearly exclusively in the outer leaflet of the MFGM, resemble host receptor analogs that bind to specific pathogen virulence factors and inhibit them from binding to their intestinal target receptors [25].

The outer portion of the MFGM is composed of a bilayer of the apical plasma membrane of the milk-secreting mammary epithelial cell from which it was derived. Next to this outer bilayer is a phospholipid-coated monolayer with associated proteins and variable amounts of other cellular material, including cytoplasmic assemblies of enzymes, mRNAs, and intracellular vesicles. The unique structural and compositional properties of MFGs, including their membranes, suggest that they are not only simply passive lipid transport vehicles but likely also communicate information about maternal factors [26], act as self-contained “metabolic systems” that can dynamically modulate their cargo [27], may influence neonatal intestinal bacteria [28], and possibly contribute to neonatal GI tract development [28] and cellular communication [29]. The potential for physical and functional interactions among these components is highlighted in a later section of this report.

The lipid concentrations and fatty acid profiles are notably variable within milk [17] and are associated with a variety of factors such as circadian rhythm [30], time within feed [31], time since last feed [31], maternal diet [19, 32, 33], and maternal adiposity [34]. Whether these naturally occurring differences impact infant growth, behavior, and development is not known. Human milk lipid content and constituents may differ between the milks of lactating parents of preterm and term infants, but this finding is not consistent, and it is possible that some methods used to determine lipid concentrations are insufficient for this purpose [20]. Some, but not all, studies suggest that human milk lipids increase with time postpartum [31, 35, 36] and decrease following Holder pasteurization (Text Box 3).

Text Box 3 **Lipids in human milk: selected research gaps.**

• Do diurnal and within-feed shifts in milk fat impact infant behaviors (e.g., appetite, milk intake, and sleep)?
• Are the benefits of MFG components similar if delivered without the MFG matrix?
• How does the size distribution of MFGs influence their digestibility and other functions in the infant GI tract?
• What is the role of cholesterol and other minor lipids in human milk, and are there acute and/or long-term impacts of not consuming these substances during early life?
• Does variation in human milk fatty acid profile have implications for infants, and are these implications dependent upon the context?
• Does variation in human milk lipid content due to the maternal adiposity level communicate environmental and/or metabolic information to the developing infant?

Alt-text: Text Box 3

#### Proteins and peptides

Human milk provides highly digestible proteins and peptides with an ideal balance of essential amino acids to support infant protein synthesis and growth. Milk protein concentrations are collectively highest in colostrum (∼20 g/L) and then stabilize (∼10 g/L) [37, 38]. Concentrations are higher in preterm than term milk—at least during the first month postpartum—and do not appear to vary with maternal diet or body fat percentage [19]. Recent evidence suggests that the average human milk protein concentrations vary substantially across populations [21], and the source of this variation is not known.

In addition to meeting the infant’s basic dietary protein and energy needs, human milk also contains myriad individual biologically active proteins that play extensive roles in protection and communication. For example, secretory immunoglobulin A (sIgA), the most abundant immunoglobulin in human milk, may provide unique vertical transmission of immune system education [39]. As the infant’s mucosa does not produce sIgA for some time, sIgA in milk serves a dual function by protecting newborn infants from infection by neutralizing pathogenic bacteria and simultaneously shaping the commensal GI microbial community structure [40, 41]. Secretory IgA has also been shown to affect host–microbiome interactions by modulating the colonic transit time of bound microbes and binding to antigens originating from the maternal diet [42]. Specificity of sIgA in milk reflects antecedent exposures experienced in the maternal GI tract and therefore has the capacity to communicate and react to environmental changes. As such, it is not surprising that sIgA specificity varies globally [7]—another likely example of eco-homeorhesis.

Recent advancements in “-omics” approaches have led to a much broader understanding of the many proteins in human milk. Indeed, proteomics analyses have revealed that human milk contains hundreds of unique proteins [43] with an array of functional characteristics, including enhancing nutrient absorption (e.g., bile salt–stimulated lipase [BSSL]), defending against pathogens (e.g., lactoferrin, lysozyme, immunoglobulins), shaping the infant’s immune system (e.g., cytokines), and guiding the development of the GI tract (e.g., epidermal growth factor [44] and insulin-like growth factor 1 [45]). Most identified milk proteins have defined functions when found elsewhere in the body (e.g., blood) but lack clearly defined functions in the context of the milk matrix and infant GI tract. Among these proteins is an array of proteases, antiproteases, and protease activators that collectively act to partially digest milk proteins within the mammary gland [46] and assist with digestion in the infant GI tract [47]. Proteases in milk differ in abundance and activity between term and preterm milk, as do the released peptides as products of their activities [48, 49]. The entire array of known bioactive peptides in human milk (as well as milk from other species) is cataloged in the Milk Bioactive Peptide Database [50], although the extent to which human milk proteins and peptides survive within the infant GI tract and have the potential to exert their bioactivities at GI sites of action remains mostly unknown. Nonetheless, recent research indicates that select milk proteins and peptides may be absorbed into the bloodstream where they would have the potential to act systemically, but the evidence is weak and not comprehensive.

How milk is handled after being expressed or pumped can substantially impact the bioactivity of some of its proteins and peptides. Pasteurization denatures lactoferrin, most immunoglobulins, lysozyme, BSSL, and some cytokines and growth factors [[51], [52], [53], [54], [55], [56]]. Conversely, pasteurization increases the activity of some proteases (e.g., plasmin and elastase), which increase the release of some peptides during milk storage, contribute to digestion in the infant [57], and/or increase degradation of potentially bioactive peptides. Pasteurization also changes protease activities and protein structures and thus likely digestive survival of proteins and peptide release. Cold storage (−20 °C) can also impact the bioactivity of some proteins. For example, it preserves some bioactive proteins [58], may allow continued activity of some enzymes [59], degrades some human milk proteins (e.g., lysozyme), and can cause flocculation of casein proteins [60]. Clearly, the holistic effects of human milk processing on protein and peptide interactions and survival, release of peptides in vivo, and infant health are unknown and will likely require a systems biology approach to understand [3] (Text Box 4).

Text Box 4 **Proteins and peptides in human milk: selected research gaps.**

• What are the independent and interactive functions of the many proteins and peptides found in human milk? How do these functions change during the digestion process? Are there implications of not consuming them during early life?
• Are differences in concentrations of specific milk proteins among population examples of ecohomeorhetic shifts to support optimal early-life fitness for a particular context?
• Are there implications of changes in human milk protein bioactivity to infants consuming processed (e.g., heated or frozen) milk?
• How do antimicrobial peptides target pathogenic microbes but spare commensals and symbiotes?

Alt-text: Text Box 4

### Other biologically active components

#### Human milk oligosaccharides and other milk glycans

Human milk contains an array of glycans defined as carbohydrates consisting of several monosaccharides linked via glycosidic bonds. These diverse, complex, and abundant glycans are present in their free form (human milk oligosaccharides, HMOs) and as glycoconjugates (glycans conjugated to proteins and lipids). HMOs are the third most abundant solid component in the human milk (following lactose and lipids) ranging on average from 23 g/L in colostrum to 15 g/L in mature human milk. More than 150 HMO structures have been identified, with ∼20 representing ∼90% of the HMOs in human milk [61].

Complex glycans, including HMOs, are unique human milk constituents in that they resist digestion by host enzymes as they transit the infant’s GI tract. Although they do not provide direct nourishment to the infant, they have long been known to nourish microbes living within the infant’s GI tract. For example, they serve as a food source for a specific suite of bifidobacterial species [62, 63]. They have also been shown to enhance the growth of milk-derived staphylococcal species, potentially via stimulating their use of amino acids [64]. In addition, some mimic recognition and attachment sites present on the apical surface of the enterocytes and, as a consequence, act as pathogen decoys [65]. They also have antimicrobial, antiviral, and immune modulating effects [61, 66, 67].

Several studies have reported that HMO concentrations vary across lactation and may be dependent on fixed factors such as maternal genetics [8], age, parity, ethnicity, and gestational age of the infant [66, [68], [69], [70]] and modifiable factors such as geography [71], seasonality [72], and maternal diet [73] and health [69]. Together, these findings suggest that variation in glycan profiles might communicate environment- and/or maternal-specific information to the infant to help shape the GI microbiome and immune system for optimal fitness in a specific location and/or context.

In addition to free HMOs, glycan conjugates (glycoproteins and glycolipids) are abundant in milk. For instance, many protective proteins found in the whey fraction of human milk (e.g., lactoferrin, lysozyme, and immunoglobulins) are heavily glycosylated, and their glycosylation patterns have been shown to have direct effects on pathogen binding, infectivity, and proteolytic susceptibility [74]. The glycosylation patterns of human milk lactoferrin and sIgA change over the course of lactation and may be influenced by maternal and infant illness, delivery mode, and metabolic status (e.g., gestational diabetes) [[75], [76], [77]].

#### Nonprotein, nitrogenous compounds

Human milk also delivers nonprotein nitrogen (NPN) compounds that collectively constitute ∼20%–25% of its total nitrogen content. NPN consists primarily of urea and also free amino acids, small peptides, glucosamine, choline, creatine, creatinine, uric acid, ammonia, nucleic acids, nucleotides, and polyamines [78]. Although AI levels have been established for choline, which is considered an essential “vitamin-like” compound [79], the remaining NPN components are considered nonessential. However, these molecules may provide the infant with metabolic and protective functions—at least indirectly. For example, urea (which makes up ∼50% of the NPN in human milk) may contribute to nitrogen homeostasis of the breastfed infant’s GI microbiome [66].

#### Hormones

Human milk also delivers hormones (e.g., cortisol, melatonin, leptin, adiponectin, and insulin) [[80], [81], [82]] that may contribute to indirectly nourishing the infant (e.g., impact of insulin on glucose absorption) as well as communicating information relative to metabolic and environmental conditions important for acute and long-term survival. For example, concentrations of melatonin and the amino acid tryptophan exhibit a circadian rhythm with undetectable concentrations in the day but high concentrations at night; this may communicate the time of day to the recipient infant [83]. In contrast, the glucocorticoid cortisol, which may be involved in behavioral programming, exhibits a circadian rhythm with higher concentrations in the morning [83]. Studies in humans and rhesus macaques showed that milk cortisol concentration is correlated with the maternal body size, parity [84], maternal circulating cortisol [85], and maternal stress [86]. Several findings suggest that variation in human milk cortisol concentrations aids in programing behavioral traits and growth outcomes in the recipient offspring [87]. In nonhuman primates, milk cortisol concentration positively correlates with nervousness, less confident temperament, and higher weight gain in the offspring when controlling for milk energy [83]. The association of milk cortisol with offspring confidence differs by the infant’s sex [85]. Similar findings have been detected in human infants, where milk cortisol is positively associated with more “negative affectivity” such as fear, sadness, and discomfort [88] and higher infant fear reactivity in a laboratory setting in only biologically female offspring [89]. Clearly, however, these correlations could also be due to environmental exposures or the impact of the lactating parent (beyond milk), and more research is needed to tease this apart. Human milk cortisol concentrations have also been positively associated with infant body fat levels [90] and with the change in BMI percentile over the first 2 y of life [91]. Although more research is needed to confirm these observations, it is possible that varying human milk cortisol concentrations may communicate to the infant the best way to thrive in the particular physical and social environment into which they were born—another possible example of a context-specific ecohomeorhetic shift in milk composition.

#### Flavor or scent compounds

Flavor and scent compounds derived from the maternal diet are also present in human milk. These compounds may prepare the infant for the flavors of the cultural diet and impact later feeding behavior and food acceptance [92, 93]. This specific mode of maternal-infant communication is discussed in more depth in the report from BEGIN WG 1 [2].

#### Xenobiotics

Human milk can also contain a multitude of exogenously produced substances derived from the environment collectively referred to as xenobiotics. Some of these molecules may play a role in protection or communicating information on the environment to the infant. For example, many secondary plant metabolites including antioxidant molecules and biologically active components of animal-based foods that are consumed appear in human milk [33, 94]. Although it is likely that these dietary components impact infant health similar to adult health, very little research has been conducted on this topic. Environmental pollutants, drugs, and toxicants can also be found in human milk. In a recent study conducted in Uganda, several persistent organic pollutants (POPs; e.g., brominated flame retardants and polychlorinated biphenyls) were detected in milk produced by relatively healthy individuals. Although the levels were somewhat lower than those reported previously in other countries [95], infant consumption of dioxins exceeded tolerable doses put forth by the World Health Organization [96]. The acute and long-term impacts of these compounds on infant health are poorly understood but may be important at high doses. In a study conducted in Norway, concentrations of some environmental contaminants in human milk were associated with the variation in infant fecal microbiome [97], although it is unknown if this represents a causal association. Arsenic, lead, mercury, and cadmium can also be detected in milk produced by individuals living in regions with elevated environmental loads of these metals [98]. Many prescription and recreational drugs can also be incorporated into milk. For example, the main psychoactive component of cannabis (tetrahydrocannabinol [THC]) can be detected in milk produced by its users [99], as well as in the case of alcohol [100]. This is particularly important because cannabis and alcohol are the 2 most commonly used recreational drugs during breastfeeding. It is noteworthy, however, that very little research has been conducted to evaluate the impact of maternal cannabis use on human milk composition and infant outcomes. Methamphetamine is also found in milk produced by users [101]. Additional information related to maternal drug and alcohol use and its impacts on milk composition and infant health and wellbeing can be found in a report prepared by BEGIN WG 3 [3]. Substantial research is warranted to understand the impacts of these compounds on infant health, particularly as related to cannabis, which is now legal in many US states (Text Box 5).

Text Box 5 **Complex, biologically active components and cells in human milk: selected research gaps.**

• Are the effects of individual HMOs in milk impacted by the presence or absence of other HMOs in milk?
• How does the glycan composition of glycoproteins and glycolipids exert their immunological functions? How do maternal diet, genetics, and environment influence their glycosylation compositions?
• Does infant genotype influence response to various HMOs in milk?
• How do HMOs interact with the infant’s immune system and intestinal epithelia?
• What are the functions of the many NPN compounds in milk?
• Do hormones and growth factors in milk communicate information regarding the maternal metabolic status to the infant?
• How does the infant’s immune system communicate with the maternal immune system to alter the composition and amount of immune protective molecules in milk?
• Are there impacts of xenobiotics (e.g., cannabinoids and environmental toxicants) on infant growth, development, and/or behavior?

Alt-text: Text Box 5

### Cells

#### Maternal cells

The maternal-derived cells in human milk are highly heterogeneous and include epithelial cells (ductal and alveolar, luminal–epithelial, and myoepithelial), stem and progenitor cells, and blood-derived leukocytes. Leukocytes include granulocytes and mononuclear leukocytes, such as lymphocytes, monocytes, and macrophages. The cell content of human milk at any lactation stage is highly variable, ranging from roughly 10,000 to 13,000,000 cells/mL with the highest concentration in colostrum and early milk compared with mature milk. It is unknown if the higher cell count observed in early lactation is because milk is more concentrated (less dilute) or is due to other factors such as physiologic differences or breastfeeding patterns.

Human milk cellular heterogeneity and cell composition are influenced by many factors, as described in another report in this series [2]. For example, leukocytes are the predominant cells in colostrum and early milk, while some reports suggest that mammary epithelial cells are the predominant cell types in mature milk [102, 103], although this finding is not consistent [104]. The presence of leukocytes is proposed to protect the mammary gland from infection during lactation but they also may respond to the health of the infant as concentrations increase during maternal or infant infection [102, 105, 106]. For example, these cells respond to viral antigens in culture with increased proliferation rate and altered expression of IL-6, IL-17A, interferon-gamma, and tumor necrosis factor-alpha, demonstrating potential roles to deliver protection in the form of innate immunity to the infant [107].

Other functions of human milk cells are not well understood but are thought to be involved in communication. For example, stem cells may deliver complex epigenetic programming resulting from a constellation of a lactating parent’s genetic, dietary, and environmental inputs [108, 109]. Animal models show that these cells are absorbed into the infant’s circulation and are detectable in the infant’s tissue after only a short duration of breastfeeding [108, 110]. Maternal stem cells may communicate these factors to the breastfed infant, although substantial research is needed to confirm this possibility (Text Box 6).

Text Box 6 **Cells in human milk: selected research gaps.**

• Which maternally derived (host) cell types are in milk?
• How does the maternal cell profile change over lactation?
• Does the maternal cell profile in human milk respond to maternal and/or infant illness and, if so, what are the implications to maternal and infant health?
• If stem cells are present, what are their functions—particularly in terms of communicating information to the infant?
• What are the origins of the microbes in human milk?
• Do human milk microbes contribute to the vitamin or other nutrient needs of the infant?
• Do inherent bacteria in milk help train the infant’s immune system to tolerate (or not) particular microbes?
• To what extent do human milk microbes colonize the infant’s GI tract, and how do they interact with other milk components (e.g., HMOs) in this regard?
• What are the implications of variation in the human milk microbe profiles for maternal and infant health?

Alt-text: Text Box 6

#### Microbes

Once considered sterile, experts now agree that milk contains a rich microbial community [[111], [112], [113]]. In addition to their likely importance in colonizing the infant GI tract, microbial communities in human milk might communicate which microbial taxa should be tolerated vs. destroyed. Although purely speculative, this theory supports the concept of eco-homeorhesis and both the “hygiene hypothesis” and related “old friends hypothesis,” which posit that early-life exposure to microorganisms protects infants against allergic disease [114] and early-life exposure to microorganisms with which we co-evolved is needed to develop immunologic tolerance so that these organisms do not evoke unhealthy inflammatory responses [115], respectively. Because many GI microbes can also supply an array of essential nutrients (e.g., vitamin K) and increase the absorbable sources of energy (e.g., short-chain fatty acids), their presence in milk might contribute directly to nourishing the infant. This possibility is expanded upon later in this manuscript.

Milk’s microbial profiles vary greatly among individuals and globally [6], and this variation might be related to diet [116], maternal body weight [117], other milk components such as amino acids and HMOs [21, 104], and a variety of other factors such as delivery mode [117], time postpartum [117], household composition (e.g., number of individuals living together) [118], and feeding/parenting practices [119, 120]. Whether this variation impacts infant health is not known, although it is likely that the milk microbiome directly assists in colonizing the infant’s GI tract [6, 121, 122].

### Extracellular Vesicles

Recent attention has also been paid to extracellular vesicles (EVs) in human milk [123]. EVs can contain DNA, RNA, proteins, microRNAs (miRNAs), and lipids and can originate from a variety of cell types, both eukaryotic and microbial [124]. Emerging data suggest that human milk miRNA encapsulated within EVs is relatively resistant to digestion and may have biological impacts on infant cells both in the intestine and systemically [[124], [125], [126], [127]]. As such, they may serve as a mode of epigenetic communication from the lactating parent to the infant [[126], [127], [128]]. For example, concentrations of specific miRNAs in human milk that may impact metabolism were shown to correlate with maternal lifetime stressors and negative events [129], suggesting that miRNA in human milk may represent an avenue whereby intergenerational trauma and maternal stress are communicated to the infant, in turn potentially impacting the infant metabolome. Considerable research is needed, however, to test this hypothesis.

### Milk as a Biological System

Most constituents in human milk likely play direct or indirect roles in nourishing and protecting the infant and also in providing customized communication that helps the infant thrive in a particular culture, environment, and genetic predisposition. It is likely, however, that the cumulative impact of human milk components on infant health is greater than the sum of the impacts of each component. Indeed, human milk is a biological system—itself part of a larger ecology comprising the lactating parent, infant, and their shared environment.

### Defining what is meant by “interaction”

**FIGURE 3:**
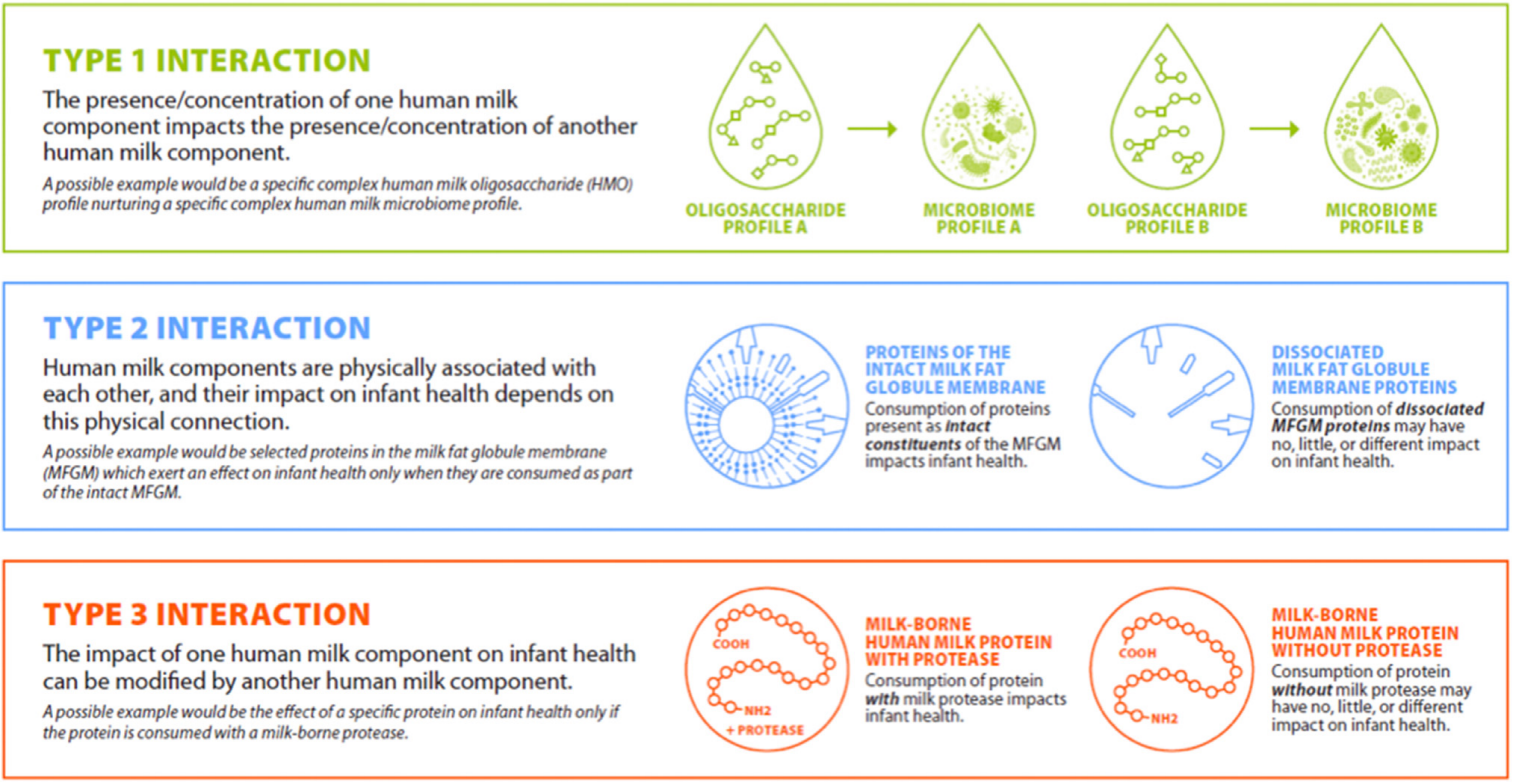
Illustration of 3 possible scenarios whereby milk components might “interact” in the mammary gland (type 1 interaction) or in the infant (type 2 and type 3 interactions) and thus impact milk composition and/or infant health.

For human milk to function as a biological system, its components must work interactively to improve infant health. Here, we provide several ways in which milk components might “interact” and then provide 3 case studies outlining possible examples of milk behaving as a biological system (Figure 3).

• Type 1 interaction: The presence or concentration of one human milk component (“component A”) impacts the presence or concentration of another (“component B”), respectively.
• Type 2 interaction: Human milk components are physically associated with each other, and their impact on infant health depends on this physical connection.
• Type 3 interaction: The impact of one human milk component (“component A”) on infant health can be modified by another (“component B”) such that the effect of a certain level of component A on the infant depends on the accompanying presence or level of component B.

#### Case example 1: interactions within and between the milk micronutriome and milk microbiome

Very little is known about whether micronutrients interact with themselves and/or other milk components to influence their individual or collective impact on infant health. This dearth of knowledge is in large part due to the fact that, until relatively recently, most micronutrients in human milk had to be analyzed individually. Now, however, with the application of methods such as liquid chromatography–mass spectrometry (LC-MS) for vitamin analysis and ICP-MS for mineral analysis, it is possible to measure many micronutrients (which we refer to as the micronutriome) simultaneously in very small volumes. Thus, data are slowly accumulating on the concentrations of multiple micronutrients in human milk samples and potential interrelationships among them and other milk components. Nonetheless, it is often difficult to evaluate whether statistical correlations between and among milk nutrients and other components are causal in nature because nutrient deficiencies tend to occur together. For example, low levels of several nutrients are often found in milk produced by females consuming diets low in animal-source foods, but it is impossible at this time to determine if that is simply due to the overall poor diet or interactions of the nutrients in the mammary gland. Similarly, in a recently published report that attempted to determine associations among milk micronutrients and macronutrients, many significant correlations were described, but again, it is impossible to know from these types of studies if these associations are causal in nature [15]. It is not surprising that concentrations of the fat-soluble vitamin were correlated with each other and total lipids because of their lipophilic nature (possibly a type 1 interaction). However, these associations may also reflect a type 3 interaction, as the ability of the infant to absorb fat-soluble vitamins is dependent on sufficient lipids in the diet for chylomicron formation. Whether any of the other nutrient–nutrient correlations described by Hampel et al. [15] represent true interactions or simply artifacts of a generally poor diet remains to be determined.

Because microbes depend on their hosts for their own unique constellation of essential nutrients, it is also plausible that variation in nutrients (and HMOs) in milk impacts which microbes thrive in the mammary milieu and consequently constitutes the human milk microbiome. Evidence to support this comes, at least in part, from 2 studies by Williams et al. [104, 116] who found myriad correlations between the concentrations of macronutrients and HMOs and the relative abundance of bacterial taxa in milk and maternal dietary micronutrient and macronutrient intake. For instance, alpha-linolenic acid and arachidonic acid concentrations in milk were positively correlated with the relative abundance of *Propionibacterium*. Moossavi et al. also reported complex relationships between milk fatty acid profiles and human milk microbial community structures, suggesting that the availability of various fatty acids might nurture particular microbial profiles [120]. Similarly, Pace et al. found substantial relationships between milk-borne HMOs and lactose and variation in the milk microbiome [21]. As with the understanding of whether milk-borne nutrients interact with each other to impact their concentrations in milk, controlled dietary intervention studies will be needed to determine if these nutrient–microbe associations in human milk are causal in nature and represent type-1 interactions and true biological systems within the mammary gland or are driven by coincidental factors. In summary, it is conceivable and theoretically plausible that nutrients and microbes in human milk impact each other’s concentrations (type 1 interaction) and/or modify each other’s impacts on infant health (type 3 interaction), but very little research has rigorously examined these possibilities.

#### Case example 2: milk fat globule components

The presence of BSSL and triglycerides in the MFG represent a likely example of type 2 and type 3 interactions. In comparison to children and adults, infants have lower concentrations of lipid hydrolytic enzymes such as co-lipase–dependent lipase needed for digestion of triglycerides and, thus, would seem to be at a disadvantage in terms of being able to digest human milk lipids. However, human milk contains ∼100 mg/mL of BSSL, a lipase able to hydrolyze all 3 bonds of a triglyceride as well as a variety of substrates, including cholesterol and fat-soluble vitamin esters and phospholipids [130]. Due to its glycosylation ability, milk-borne BSSL is resistant to degradation by gastric juices and therefore reaches the small intestine where it can be activated by bile salts, in part due to its protection within MFG (type 2 interaction). In vitro studies have demonstrated that it is a combined action by gastric lipase, co-lipase–dependent pancreatic lipase from the infant, and milk-borne BSSL that leads to near complete hydrolysis of MFG triglycerides and long-chain polyunsaturated fatty acids (LCPUFAs) to free fatty acids and glycerol [131, 132]. Indeed, inactivation of BSSL by pasteurization reduces the overall lipid absorption from human milk by as much as one-third [133]. Thus, the presence of BSSL in human milk exemplifies both type 2 and type 3 interactions in which one component (BSSL) directly impacts the extent to which another component (fatty acids in the MFG) is available for utilization by the infant, but this effect requires BSSL to be physically associated with the MFG matrix. Understanding this potential complex interaction might be particularly important to elucidating the nourishing nature of human milk. In addition, because many fatty acids have antimicrobial functions, this complex interaction might better inform our understanding of human milk’s protective functions.

The presence of xanthine oxidase reductase (XOR) as part of the MFG is another example of a complex human milk component, in this case exhibiting both type 1 and type 2 interactions with other milk components such as butyrophilin, sulfhydryl oxidase, lactoperoxidase (LPO), and nitrate. XOR is a multifaceted enzyme with both structural and metabolic functions. XOR is an abundant MFG protein that forms a complex with butyrophilin, a transmembrane member of the immunoglobulin superfamily implicated in milk lipid secretion [134]. Evidence of a type 1 interaction between XOR and butyrophilin comes from transgenic mouse studies, which demonstrated that XOR modulates milk lipid secretion and influences MFG structure and protein composition, including reducing levels of butyrophilin and increasing levels of BSSL [135]. XOR enzymatic activity produces reactive oxygen and nitrogen species that have been shown to have antimicrobial and infant immune defense properties [136, 137]. As such, these interactions might have both nourishing and protective impacts on the infant.

XOR in the human mammary gland and MFGs may be found in 2 enzymatic forms: xanthine dehydrogenase (XD) and XO. XD is the predominate form of the enzyme within mammary epithelial cells, but during MFG secretion, XD is exposed to apical membrane–bound sulfhydryl oxidase (type 2 interaction), which then rapidly converts it to the XO form [138]. XO in milk is then able to generate peroxide substrates that can subsequently be converted to hypothiocyanite, a potent bactericidal compound, by LPO, another enzyme found in milk. XO also can convert milk-borne nitrate to nitrite, once again providing a substrate for LPO that then converts the nitrite to nitrogen dioxide, another bactericidal compound. Additionally, the fact that XOR resides on the inner membrane leaflet of MFG provides a structural framework whereby XOR activity may be resistant to the inactivation of proteases and, thus, maintains its antimicrobial activity within glandular structures and the infant digestive system, potentially impacting their microbial compositions and risk of infection.

#### Case example 3: proteins, proteases, and peptides

As noted above, milk contains a multitude of proteins, proteases, naturally occurring milk protein–derived peptides, and peptide hormones, and it is likely that these components interrelate with one another within the mammary gland, resulting in a type 3 interaction. For instance, milk contains an array of proteases (plasmin, cathepsins, elastase, kallikrein, thrombin, etc.) that differ in cleavage specificities [48, 50]. Moreover, for each protease, milk contains an array of protease inhibitors, protease activators, and protease activator inhibitors that control the relative abundances of the zymogen and active forms and the degree of activity of the active forms [50, 51]. Protease activation can be microlocation-specific; for example, plasmin activity appears to be higher within the casein micelle than within the whey [139]. These proteases interact with milk proteins within the mammary gland, releasing thousands of unique peptides from human milk proteins with apparent protein-level and site-specificity [140].

Protein-level specificity may also be related to the protein structure and modifications like glycosylation and microlocation (casein micelle vs. freely soluble whey proteins) [141]. Yet, this proteolytic process is controlled such that most of the milk proteins remain intact. Many of the released peptides are identical to or homologous with known bioactive peptides with antimicrobial, immunomodulatory, and antihypertensive activity [140, 142]. The milk proteases and the peptides they release likely interact with mammary epithelial cells, immune cells, and/or bacteria within milk. Yet, interactions of this nature remain unexamined.

Nonetheless, it is likely that these milk components interact, and this interaction, at least in part, is required for them to impact infant health. For instance, digestion of human milk proteins by milk proteases (and also gastric proteases) can activate milk peptide hormones via proteolytic cleavage. An example would be TGF-β in milk, which is inactive until it is exposed to low pH and protease activity (either gastric or milk proteases) in the infant’s stomach.

Milk proteins and peptides also interact with milk minerals to impact infant health (type 2 interaction). For example, lactoferrin binds iron, assisting in iron absorption and restricting its use by microbes [143]. Many peptides derived from the digestion of the casein micelle are highly phosphorylated, enabling solubilization of calcium and enhanced calcium absorption [144, 145]. In summary, milk proteins, proteases, peptides, and peptide hormones have an array of known interactions within milk and the infant—in particular, impacting nourishment and protection of the infant. Yet, much remains unknown about these interactions and their impact on early-life health, thereby warranting future investigations.

## Summary and Knowledge Gaps

In summary, human milk’s complex composition and structural matrix comprises literally thousands of molecules and cell types—many of which likely function independently and interactively to nourish, protect, and communicate complex information to infants, thereby impacting lifelong health. We posit that these putative interactions take a variety of forms including impacting each other’s concentrations in milk; physically associating with each other, which is necessary to benefit infant health and function; and modifying each other’s functions once ingested by the infant. However, almost no research has been conducted to adequately test these interactions in a rigorous manner. The report of BEGIN WG 4 [4] provides additional details and covers many of the key issues to be addressed to facilitate our ability to study human milk as a biological system.

With specific regard to the issues covered here, controlled dietary intervention studies will be needed to test the 3 types of interactions proposed in this manuscript. More importantly, understanding if and how these interactions work as part of human milk’s biological system embedded within a particular ecology to impact maternal and infant health is critical. In many cases, animal models will be needed, although it behooves human milk researchers to explore creative experimental designs to test these hypotheses in human subjects if possible. In addition, because of the complexities related to analytical approaches, multidisciplinary research teams will need to fully collaborate to elucidate how milk acts as a biological system. The complexity of human milk composition will require highly sensitive, comprehensive methodologies involving a “multi-omics” approach. Because both milk composition and infant health are greatly impacted by social, behavioral, and environmental contexts, multicohort (and likely international) approaches should be prioritized to understand whether differences observed among cohorts have adaptive consequences for human health. Finally, researchers are encouraged to assess the potential impact of biological sex and gender identity on milk composition, when possible, as nothing is known about this important relationship. Understanding the complexity of milk and its impact on infant health as a combined product of both immediate environmental and diet and evolutionary pressures within the ecology of the maternal–infant–environment context is fundamental to understanding the basics of human nutrition itself.

## Funding

The BEGIN Project was initiated by the Pediatric Growth and Nutrition Branch of the *Eunice Kennedy Shriver* National Institute of Child Health and Human Development (NICHD), the National Institutes of Health (NIH), in partnership with the Bill & Melinda Gates Foundation (BMGF) and the Academy of Nutrition and Dietetics (Academy). This supplement was supported by the *Eunice Kennedy Shriver* NICHD, the United States NIH. Support for assistance (by BioCentric, Inc.) with editing, proofing, and submitting the manuscript was also provided by the *Eunice Kennedy Shriver* NICHD.
